# Supplementary material for: A Critical View of the Application of the APEX Software (Aqueous Photochemistry of Environmentally-Occurring Xenobiotics) to Predict Photoreaction Kinetics in Surface Freshwaters
Source: Molecules. 2019 Dec 18;25(1):9. doi: 10.3390/molecules25010009 (PMC7017383; doi:10.3390/molecules25010009)
Supplement: Supplementary file 1 [file molecules-25-00009-s001.zip › 3.2.4_gcc-4.4.0.zip!/3.2.4_gcc-4.4.0/3.2.4_gcc-4.4.0/doc/octave/html/interpreter/Sparse-Functions.html]

Sparse Functions - Untitled


Next: Return Types of Operators and Functions,
Up: Operators and Functions


---

##### 21.1.4.1 Sparse Functions

An important consideration in the use of the sparse functions of
Octave is that many of the internal functions of Octave, such as
diag, cannot accept sparse matrices as an input. The sparse
implementation in Octave therefore uses the dispatch
function to overload the normal Octave functions with equivalent
functions that work with sparse matrices. However, at any time the
sparse matrix specific version of the function can be used by
explicitly calling its function name.

The table below lists all of the sparse functions of Octave. Note that
the names of the
specific sparse forms of the functions are typically the same as
the general versions with a sp prefix. In the table below, and the
rest of this article the specific sparse versions of the functions are
used.

Generate sparse matrices:: spalloc, spdiags, speye, sprand, sprandn, sprandsym Sparse matrix conversion:: full, sparse, spconvert Manipulate sparse matrices: issparse, nnz, nonzeros, nzmax, spfun, spones, spy Graph Theory:: etree, etreeplot, gplot, treeplot Sparse matrix reordering:: amd, ccolamd, colamd, colperm, csymamd, dmperm, symamd, randperm, symrcm Linear algebra:: condest, eigs, matrix\_type, normest, sprank, spaugment, svds Iterative techniques:: luinc, pcg, pcr Miscellaneous:: spparms, symbfact, spstats

In addition all of the standard Octave mapper functions (i.e., basic
math functions that take a single argument) such as abs, etc.
can accept sparse matrices. The reader is referred to the documentation
supplied with these functions within Octave itself for further
details.
